# Fluoroquinolone-resistant *Salmonella* sp. in Carcasses

**DOI:** 10.3201/eid1202.050629

**Published:** 2006-02

**Authors:** Yu-Chih Wang, Kuang-Sheng Yeh, Chao-Chin Chang, Shih-Ling Hsuan, Ter-Hsin Chen

**Affiliations:** * National Chung Hsing University, Taichung, Taiwan;; † Taipei Medical University, Taipei, Taiwan

**Keywords:** Salmonella, fluoroquinolone, Taiwan, letter

**To the Editor:** Fluoroquinolone (FQ)-resistant *Salmonella* has been isolated from patients in Taiwan (*1**–**7*). Recently, a report further indicated that several patients were infected with *Salmonella enterica* serovar Schwarzengrund with high-level FQ resistance (*1*). *S*. Schwarzengrund has never been isolated from food animals in Taiwan.

We report the isolation of FQ-resistant strains from pork and broiler carcasses sampled from 2000 to 2003: 27 in 2000, 3 in 2001, 4 in 2002, and 2 in 2003. These isolates made up 18.85% of the 191 *Salmonella* strains obtained from pork and broiler carcasses in the study period. Of these isolates, 16 FQ-resistant *S*. Schwarzengrund strains were further analyzed to elucidate the possible mechanism of FQ resistance. Ciprofloxacin MIC levels in these isolates ranged from 4 to 16 μg/mL, and all had high-level nalidixic acid resistance (>1,024 μg/mL). All of the 16 investigated strains displayed mutations possibly associated with high-level FQ resistance. The mutation sites included 2 sites (Ser83Phe and Asp87Gly) in the quinolone resistance–determining region (QRDR) of *gyrA*, 2 sites (Thr57Ser and Ser80Arg) in the QRDR of *parC*, and 1 site (Ser458Pro) in the QRDR of *parE*, respectively. Four strains had mutations in the QRDR of *gyrA* and *parC* only but not in the QRDR of *parE* (Table).

**Table:** Characteristics of ciprofloxacin-resistant *Salmonella enterica* serovar Schwarzengrund strains from carcasses*†

Strain no.	Origin*	Year isolated	Antimicrobial drug resistance profile	Quinolone MICs (μg/mL)				Substitutions in QRDR‡		
				NAL	FLU	ENR	CIP	*gyrA*	*parC*	*parE*
A5	B, M	2000	CmSxtTc	1,024	512	32	8	Ser83Phe; Asp87Gly	Thr57Ser; Ser80Arg	Ser458Pro
A16	P, E	2000	ApCmNSxtTc	2,048	512	32	8	Ser83Phe; Asp87Gly	Thr57Ser; Ser80Arg	Ser458Pro
A17	P, E	2000	ApCmNSxtTc	2,048	512	32	16	Ser83Phe; Asp87Gly	Thr57Ser; Ser80Arg	Ser458Pro
A18	P, E	2000	ApCmNSxtTc	2,048	512	32	16	Ser83Phe; Asp87Gly	Thr57Ser; Ser80Arg	Ser458Pro
A19	P, E	2000	ApCmCnNSxtTc	1,024	512	32	8	Ser83Phe; Asp87Gly	Thr57Ser; Ser80Arg	Ser458Pro
A20	P, E	2000	ApCmNSxtTc	2,048	512	32	8	Ser83Phe; Asp87Gly	Thr57Ser; Ser80Arg	Ser458Pro
A29	B, S	2000	CmNSxtTc	1,024	512	32	8	Ser83Phe; Asp87Gly	Thr57Ser; Ser80Arg	Ser458Pro
A36	B, S	2000	ApCmSxtTc	1,024	512	32	8	Ser83Phe; Asp87Gly	Thr57Ser; Ser80Arg	Ser458Pro
A41	P, S	2000	ApCmCnNSxtTc	1,024	512	32	16	Ser83Phe; Asp87Gly	Thr57Ser; Ser80Arg	Ser458Pro
A45	P, S	2000	ApCmNSxtTc	1,024	512	32	16	Ser83Phe; Asp87Gly	Thr57Ser; Ser80Arg	
A51	P, S	2000	ApCmCnNSxtTc	1,024	512	16	4	Ser83Phe; Asp87Gly	Thr57Ser; Ser80Arg	
A56	B, M	2000	ApCmCnNSxtTc	2,048	512	64	16	Ser83Phe; Asp87Gly	Thr57Ser; Ser80Arg	
A61	P, S	2000	CmSxtTc	1,024	512	32	8	Ser83Phe; Asp87Gly	Thr57Ser; Ser80Arg	Ser458Pro
A62	P, S	2000	ApCmCnSxtTc	2,048	512	64	16	Ser83Phe; Asp87Gly	Thr57Ser; Ser80Arg	
B16	P, E	2001	ApCmCnCroTc	2,048	512	32	16	Ser83Phe; Asp87Gly	Thr57Ser; Ser80Arg	Ser458Pro
B73	P, N	2003	ApCmCnNSxtTc	2,048	512	32	16	Ser83Phe; Asp87Gly	Thr57Ser; Ser80Arg	Ser458Pro

*QRDR, quinolone resistance–determining region; B, broiler; M, middle Taiwan; P, pork; E, east Taiwan; S, south Taiwan; N, north Taiwan. †Antimicrobial agents are ampicillin (Ap), chloramphenicol (Cm), ciprofloxacin (CIP), enrofloxacin (ENR), flumequine (FLU), gentamicin (Cn), ceftriaxone (Cro), nalidixic acid (NAL),neomycin (N), trimethoprim/sulfamethoxazole (Sxt), and tetracycline (Tc). ‡No *gyrB* substitutions were detected.

In conclusion, high-level FQ resistance was detected in *S*. Schwarzengrund isolated from pork and chicken in Taiwan. Specific mutation sites of *gyrA*, *parC*, and *parE* were associated with high-level FQ resistance in all the isolates investigated. Our results warrant further investigation of the public health consequences of FQ use in food animals in Taiwan.
